# RNA sequencing of corneas from two keratoconus patient groups identifies potential biomarkers and decreased NRF2-antioxidant responses

**DOI:** 10.1038/s41598-020-66735-x

**Published:** 2020-06-18

**Authors:** Vishal Shinde, Nan Hu, Alka Mahale, George Maiti, Yassine Daoud, Charles G. Eberhart, Azza Maktabi, Albert S. Jun, Samar A. Al-Swailem, Shukti Chakravarti

**Affiliations:** 1 Department of Ophthalmology, NYU Langone Medical Center, New York, NY USA; 2 King Khaled Eye Specialist Hospital, Riyadh, Saudi Arabia; 3 Wilmer Eye Institute, Johns Hopkins University School of Medicine, Baltimore, MD USA; 4 Ophthalmology and Oncology Johns Hopkins University School of Medicine, Baltimore, MD USA; 5 Department of Pathology, NYU Langone Medical Center, New York, NY USA

**Keywords:** Gene expression analysis, Gene regulation, Eye diseases, Corneal diseases

## Abstract

Keratoconus is a highly prevalent (1 in 2000), genetically complex and multifactorial, degenerative disease of the cornea whose pathogenesis and underlying transcriptomic changes are poorly understood. To identify disease-specific changes and gene expression networks, we performed next generation RNA sequencing from individual corneas of two distinct patient populations - one from the Middle East, as keratoconus is particularly severe in this group, and the second from an African American population in the United States. We conducted a case: control RNA sequencing study of 7 African American, 12 Middle Eastern subjects, and 7 controls. A Principal Component Analysis of all expressed genes was used to ascertain differences between samples. Differentially expressed genes were identified using Cuffdiff and DESeq2 analyses, and identification of over-represented signaling pathways by Ingenuity Pathway Analysis. Although separated by geography and ancestry, key commonalities in the two patient transcriptomes speak of disease - intrinsic gene expression networks. We identified an overwhelming decrease in the expression of anti-oxidant genes regulated by NRF2 and those of the acute phase and tissue injury response pathways, in both patient groups. Concordantly, NRF2 immunofluorescence staining was decreased in patient corneas, while KEAP1, which helps to degrade NRF2, was increased. Diminished NRF2 signaling raises the possibility of NRF2 activators as future treatment strategies in keratoconus. The African American patient group showed increases in extracellular matrix transcripts that may be due to underlying profibrogenic changes in this group. Transcripts increased across all patient samples include Thrombospondin 2 (*THBS2*), encoding a matricellular protein, and cellular proteins, *GAS1*, *CA*SR and OTOP2, and are promising biomarker candidates. Our approach of analyzing transcriptomic data from different populations and patient groups will help to develop signatures and biomarkers for keratoconus subtypes. Further, RNA sequence data on individual patients obtained from multiple studies may lead to a core keratoconus signature of deregulated genes and a better understanding of its pathogenesis.

## Introduction

Keratoconus (KCN) is a condition where the cornea develops bilateral ectasia, becomes progressively thin and protrudes conically. The patient develops astigmatism, myopia, corneal scarring, with eventual loss of vision^1–5^. The major form of keratoconus is asyndromic, where the cornea alone is affected. However, syndromic types of KCN also exist, and are associated with Down, Leber congenital amaurosis, Turner, Marfan and Ehlers-Danlos syndromes^6^. Isolated KCN affects individuals in adolescence with an incidence and prevalence of 13.3/100,000 and 265/100,000, respectively, in subjects of European ancestry^7,8^, with a higher reported prevalence in populations from India, Pakistan and Saudi Arabia^9,10^. The prognosis of KCN depends on the severity and management of the disease^11^. There are no curative treatments but UV crosslinking of collagen fibrils is used to slow the progressive loss of corneal shape^12,13^. Other disease management options include contact lens use, corneal inserts or “intacs” and, in severe cases, cornea transplantation.

Both the corneal epithelium and stroma harbor pathogenic changes in KCN, and these have been considered as the initial causal steps^14–16^. Microscopy and biochemical studies reported various changes as important disease-specific pathologies. The epithelium appears thickened in some studies, with breaks in Bowman’s membrane. The thinner stroma shows reduced cell density^17–19^ as well as altered ultrastructure of collagen fibrils and proteoglycans^20,21^. Studies also cite increases in acid esterases, acid phosphatases, acid lipases, cathepsin B and G, matrix metalloproteinases and decreases in tissue inhibitors of metallo-proteases (TIMP)^22–27^. Recent proteomic studies, including from this laboratory, show changes consistent with loss of epithelial integrity, altered stromal ECM proteins, reduced hydroxylated collagens, and changes in integrated stress response and TGFß signaling^28–32^. These diverse changes are consistent with KCN being inherited as a complex multifactorial disease with genetic and environmental influences that ultimately cause loss of corneal integrity^6,33,34^. Genetic etiologies have been pursued through genome-wide association studies of central corneal thickness and keratoconus, implicating *VSX1*, *LOX*, *ZNF469*, *SOD1*, *TGFBI*, *FOXO1*, *FNDC3B*, *ZFN469*, *COL5A1* and *AKAP13*^6,33,35^. A number of environmental stressors, eye rubbing, contact lens wear, dry climate and UV exposure, are suspected to induce or exacerbate the disease as well^36–38^. In addition, biomarkers for keratoconus can help early diagnosis or predict disease progression. Thus, a recent study has identified prolactin-inducible protein as a promising predictive biomarker for keratoconus^39^. RNA sequence data can serve a valuable starting point for development of additional biomarkers for keratoconus.

We opted to use next generation sequencing of total RNA from patient corneas to provide an unbiased and comprehensive view of the transcriptome and disease pathogenesis. To date, two studies have reported transcriptomic analyses of corneas obtained during transplantation^40,41^. The first, compared KCN with non-KCN corneas in patients undergoing grafting for other conditions, and the second compared a smaller set of KCN corneas with control donor corneas from deceased individuals. Although these studies cited changes in members of the WNT, TGFß and ECM pathways, deeper regulatory networks were not identified.

Here we performed transcriptomic analyses of all protein-coding genes on patients from two distinct populations – African American and Middle Eastern subjects from United States and Saudi Arabia respectively. We compared these with non-diseased donor corneas, to identify common, generalizable pathogenic features. Keratoconus is particularly severe in the Middle East^9^, and therefore, we included patients from this region to increase the likelihood of finding major pathogenic changes. We identified genes related to acute phase tissue injury and NRF2-regulated oxidative stress response as core regulatory networks disrupted in keratoconus. We observed upregulations in ECM genes primarily in African American patients, suggesting underlying profibrogenic changes in this group. We detected a few genes, upregulated across all samples, which include matricellular *THBS2*, and cellular protein-encoding genes *GAS1*, *CASR* and *OTOP2*.

## Results

### Donor and patient samples

We recruited keratoconus patients of African American ancestry (KC) from Baltimore (17–69 years old) and of Middle Eastern ancestry (KJ) from Saudi Arabia (18–36 years old). **(**Supplemental Table S1). As controls for the KC samples, we used corneas from deceased African American donors (25–75 years old) for the KC samples. For the KJ corneas, we used European American donor corneas for two reasons. First, corneal tissue donation in Saudi Arabia is extremely rare making them unavailable for research^42^. Second, Middle Eastern individuals are genetically much closer to individuals of European than African ancestry^43^.

**Figure 1:**
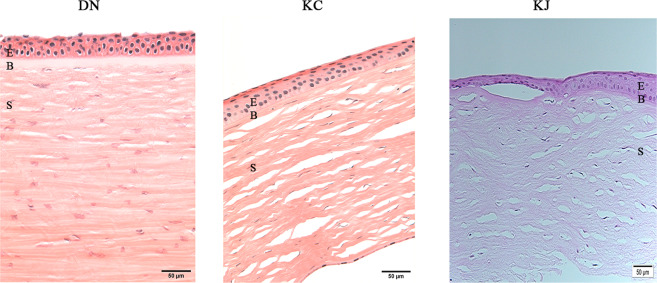
Histology of patient and donor cornea sections. The figure shows similar retention of epithelial layers in DN and KC corneas, only KJ sample showed irregular and thickened epithelial cell layer in H&E staining of paraffin embedded sections. KC: keratoconus cornea from an African American patient; KJ: keratoconus cornea from a Middle Eastern patient; E: Stratified squamous epithelium; B: Bowman’s Layer; S: stroma; K: keratocytes.

A majority of the samples retained the epithelial layer but were missing all or part of the single-cell layered endothelium. Paraffin sections stained with H&E showed the corneas to have an attached epithelium and a mostly intact stroma. The KJ sample showed an irregular and slightly thickened epithelium, but otherwise intact epithelia and stroma (Fig. 1). This indicated that the quality and epithelial retention were similar for KCN and donor corneas, although the former were placed immediately at −80 °C or processed for RNA extraction, while donor corneas were extracted one week after their receipt in Optisol at 4 °C. Further analyses of RNA quality, read depth, yield of transcripts and genes showed no difference between donor and KC samples indicating that Optisol-storage did not have a detectable negative impact (discussed below).

### RNA quality, sequence quality and patient-donor differences

The average RNA yield and RIN value among all 36 samples was 1.88 μg and 8.02, respectively (Supplemental Table S2). Across all samples, we detected 15,197–17,950 transcripts encoded by 11,286–13,025 unique protein-coding genes per sample with FPKM $\ge$ 1, and 6,900–9,155 transcripts encoded by 6,507–8,559 unique protein coding genes per sample at FPKM $\ge$ 5 (Table 1). For this study, we focused only on coding transcripts to identify gene-level changes between the patient groups. The data have been deposited in Gene Expression Omnibus at NCBI and are accessible through GEO Series accession number GSE151631 (https://www.ncbi.nlm.nih.gov/geo/query/acc.cgi?acc= GSE151631). To assess sequencing and analysis accuracy, the same RNA preparation for each of the eight samples (7 KC, 1 donor) was sequenced twice and the results show very high correlation between these technical replicates (Supplemental Fig. S1).

**Table 1:** Transcript and gene counts for each patient and control sample.

Sample ID	# Genes with FPKM ≥ 1	# Genes with FPKM ≥ 5	# Transcripts with FPKM ≥ 1	# Transcripts with FPKM ≥ 5
LE2C	12,111	7,648	16,734	8,152
LE4C	11,286	6,613	15,197	6,986
LE6C	11,876	7,615	16,371	8,075
DN5487	11,411	6,507	15,440	6,900
DN5511	11,601	7,000	15,972	7,565
DN76411	11,864	7,182	16,279	7,695
DN76507	11,799	7,321	16,228	7,923
KC276_2	12,265	7,843	16,971	8,457
KC366_2	11,591	7,409	15,860	7,918
KC369_2	11,761	7,768	16,240	8,258
KC388_2	11,476	7,457	15,839	8,089
KC395_2	12,442	8,240	17,242	8,738
KC400_2	11,994	7,822	16,509	8,291
KC406_2	12,948	8,462	17,887	9,057
KJ04	12,093	7,751	16,665	8,373
KJ05	11,740	7,868	16,405	8,540
KJ06	11,860	7,867	16,295	8,393
KJ09	11,784	7,563	16,437	8,119
KJ10	12,132	7,773	16,970	8,391
KJ11	11,753	7,659	16,417	8,339
KJ12	12,137	8,056	16,946	8,648
KJ13	11,859	7,972	16,519	8,566
KJ14	11,946	7,971	16,684	8,534
KJ17	11,862	7,925	16,600	8,529
KJ22	11,873	7,767	16,775	8,499
KJ25	12,094	7,925	16,758	8,547
***Samples not used for differential expression analysis***				
DN373	12,179	7,698	16,976	8,109
DN401	11,749	7,548	16,315	8,074
DN401_2	11,743	7,587	16,337	8,132
KC276	12,342	8,010	17,102	8,583
KC366	11,727	7,608	16,063	8,075
KC369	11,861	7,932	16,394	8,428
KC388	11,722	7,974	16,173	8,637
KC395	12,491	8,310	17,306	8,842
KC400	12,076	8,012	16,711	8,507
KC406	13,025	8,559	17,950	9,155

**Figure 2:**
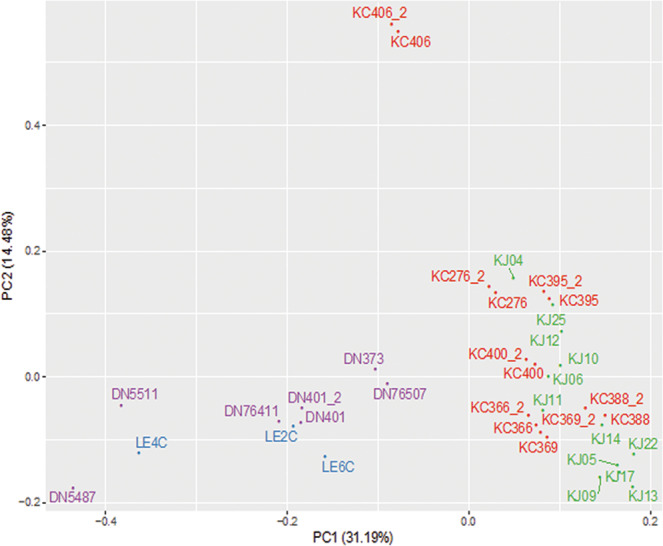
Principal component analysis (PCA) of patient and donor samples. PCA was performed on 19 Keratoconus (7 KC and 12 KJ) and 10 control (7 DN and 3 LE) corneas using 10,652 genes with FPKM value ≥ 5 in at least one sample. Technical duplicates of 7 KC and 1 DN samples (KC*_2 and DN401_2) appear close together. The major separation along PC1 was between KCN and controls. KC and KJ samples cluster together along PC1. African American donor (DN) and KC patient samples are labeled purple and red, respectively; Caucasian donors (LE) are in blue and Middle Eastern patients (KJ) in green. Euclidean distance between samples in a pairwise comparison of samples in PC1 and PC2 is shown in Supplemental Table S3.

To determine the relationships between all 36 samples, we conducted principal component analysis (PCA) using 10,652 expressed genes with FPKM $\ge$ 5 in at least one sample (Fig. 2). The first principal component (PC1) accounted for 31.2% of the expression variance and separated all control healthy tissues from diseased corneal tissues. The second principal component (PC2) accounted for 14.5% of the expression variance and was largely explained by one outlier (KC406) which was nevertheless consistent with its replicate. A scree plot shows the variance as represented by each principal component (Supplemental Fig. S1B). Pairwise comparison of all samples is shown in Supplemental Table S3. The mean distance between technical duplicates is small (2.57 $\pm$ 0.97), while comparisons of cases to controls show ~30-fold larger mean distances: 74.99 $\pm$ 27.96 and 73.15 $\pm 18.36$ between the 4 DN (control) versus the 7 KC, and the 3 LE (control) versus the 12 KJ samples, respectively. Additionally, mean distances between cases and controls are 2 and 4.8 standard deviations greater than distances within cases or controls. Repeat PCA using a smaller set of 4,787 differentially expressed genes (DEG) with FPKM ${\rm{\ge }}$5 in *all* samples (see later) showed similar relationships albeit with a significantly smaller separation along the minor axis (Supplemental Fig. S2). Keratoconus associated with atopy, hay fever or vernal keratoconjunctivitis, may represent a subtype within keratoconus; in our patient groups there were 4 such cases in the KJ and one in the KC group. However, these five showed no clustering or clear separation from other cases: mean distances between the allergy and non-allergy patients was 11.55 $\pm 7.53$, and, within the non-allergy group was 14.55 $\pm 8.26.$ In addition, there were no DEGs that were significantly different between the allergy and non-allergy KCN, and larger sample size in the future may reveal gene signatures for these subtypes of KCN (Supplemental Table S4**)**.

### Differentially expressed genes (DEGs) in patient and donor groups

We tested expression differences between donor and patient corneas of 13,397 genes^44,45^, retaining those with q $\le$ 0.01, ≥2 or ≤−2-fold change and with genes expressed at FPKM $\ge$5 in at least one sample. This filtered set contained 819 (232 upregulated, 587 downregulated) and 993 (213 upregulated, 780 downregulated) unique protein-coding DEGs in the Baltimore KC and Saudi Arabian KJ groups, respectively (Supplemental Table S4). Analysis using a second independent method showed high concordance, 80–81% of the DEGs in each canonical pathway (discussed later) were identical (Supplemental Table S5**)**^46^.

**Figure 3:**
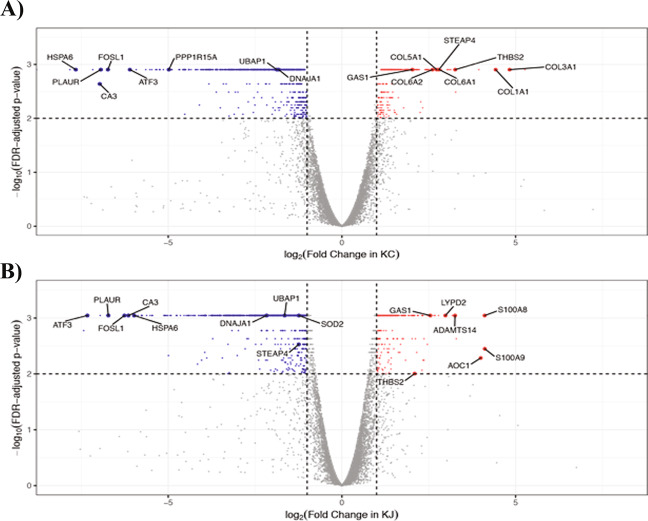
Differentially expressed genes (DEGs) in patient groups. (**A**) DEGs in KC. (**B**) DEGs in KJ. Among successfully tested genes, 8,762 and 8,990 genes have log_2_ (fold change) between −8 and 8 in KC and KJ, respectively. Down-regulated genes (q value ≤ 0.01, and fold change ≤−2, and FPKM ≥5 in control or patient groups) are labeled in blue; up-regulated genes (q value ≤ 0.01, and fold change ≥2, and FPKM ≥5 in control or patient groups) are labeled in red; genes not significantly changed are in grey.

Upon comparing the DEGs in the two patient groups, we detected 53 commonly up-regulated and 380 commonly down-regulated genes, and an additional 179 and 160 uniquely up-regulated genes in KC and KJ, respectively (Table 2). Only a few genes showed opposite expression trends in the two groups: *LGALS7*) was elevated in KJ but decreased in KC, while 12 genes (*CD34, CHST6, COLGALT2, DKK2, KDR, KERA, MAMDC2, MME, NANOS, PDGFD, RECK, STEAP4*) were decreased in KJ and increased in KC. Thus, patient corneas from two distinct populations demonstrate highly concordant differences. This is further supported by similar distributions of DEGs when categorized by cellular localization and annotated functions of the encoded proteins (Supplemental Fig. S3A,B). All DEGs with FDR adjusted *p*-value for the two KCN groups are shown in two volcano plots in Fig. 3; we detected greater similarities in down regulated than upregulated genes in the two patient groups (detailed list in Supplemental Table S6). For example, both volcano plots show significant decreases in *ATF3*, *CA3*, *FOSL1*, *PLAUR* and *UBAP1* gene expression. Many of these genes are connected to stress response as discussed later.

**Table 2:** Comparison of differentially expressed genes in African American (KC) and Middle Eastern (KJ) patients.

	Up-regulated in KJ	No change in KJ	Down-regulated in KJ
Up-regulated in KC	53	167	12
No change in KC	159	12,031	388
Down-regulated in KC	1	206	380

### Fewer similarities in most increased transcripts in the two patient groups

There are few overlaps in the ten most elevated transcripts between the two groups of samples. Most increased transcripts unique to KC corneas include 7 that encode ECM and matricellular proteins (Fig. 3A, Supplemental Table S6A). By contrast, the ten most increased DEG in KJ samples (Supplemental Table S6B) include genes that regulate inflammation (*S100A9, S100A8, LGALS9C, LCN2*), oxidative deamination of histamine and allergic response (*AOC1*), eye development and chloride channel functions (*CLIC*), and *ADAMTS14*, encoding a collagenase required for the production of assembled mature collagen fibrils^47^.

The few highly upregulated genes shared by both patient groups require further studies as these may harbor promising biomarkers for keratoconus. Thrombospondin 2 (*THBS2/TSP2*), a matricellular protein-encoding gene was significantly increased in both patient groups (Supplemental Table S6A). THBS2 is involved in cell-ECM interactions and collagen fibrillogenesis as evidenced by thin corneas in *Thbs2* null-mice^48–50^. Within the eye, it is present in the lens epithelium, injured corneal epithelium and the stroma^51^. Other significant DEGs increased in both sample sets, with FPKM $\ge 5$, include genes encoding cellular proteins that regulate growth (GAS1), Calcium sensing (CASR), exocytosis (RAB3D), cytoskeleton (KRT78) and proton-channel (OTOP2) (Fig. 3A,B, Supplemental Table S4).

In contrast, the ten most decreased DEGs (Supplemental Table S6C) show considerable overlaps between the two patient groups and point to shared pathogenesis. *SLC2A3* encodes a glucose transporter; its decrease may be upstream of nutrient deprivation-related stress. Decreased transcripts for *PLAUR*, involved in plasmin formation, *CHI3L1*, encoding a chitinase-like protein, and possibly a promoter of tissue remodeling, may both be part of a tissue injury response program. The gene encoding cyclic AMP dependent transcription factor ATF3, decreased in both groups (Fig. 3A,B, Supplemental Table S6C), regulates cell proliferation and differentiation, and is a stress response gene that interacts with JUN and FOS; additionally, related genes, *JUND* and *FOLSL1* are both downregulated in the keratoconus samples. Decreased expression of *NR4A1* encoding a steroid-retinoid hormone receptor, known to promote apoptosis may also be part of a dysregulated injury response. Other notable decreases with FPKM $\ge$ 5 in the KC cases include *SERPINB2*, *PPP1R15 A (GADD34)*, *KRT17*, *APOBEC3A*, *PTGS2*, *GADD45A*, *GADD45B*, *TM4SF1*, *NEDD9* and *PIM1*. Among the KJ samples similar decreases were seen for *PPP1R15A, PIM1*, *NEDD9* and *GADD45B* (Supplemental Table S4).

### Decreased acute phase and NRF2-mediated oxidative stress in keratoconus

**Figure 4:**
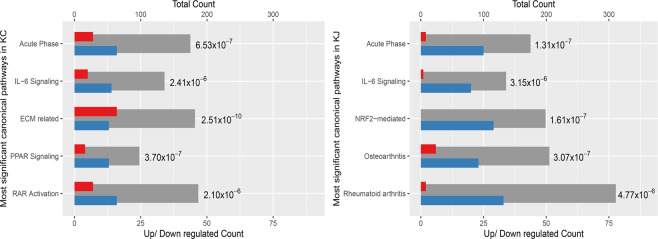
Canonical pathways significantly altered in KC and KJ. Grey bars show total number of genes in the pathway (scale on top X axis), with blue and red bars showing the numbers of down-regulated and up-regulated genes (scale on bottom X axis). The *p* value next to each bar was calculated for that pathway by the IPA Core Analysis. “Acute Phase” is the Acute Phase Response Signaling pathway; “NRF2-mediated” is NRF2-mediated Oxidative Stress Response pathway; “ECM related” is the Hepatic Fibrosis / Hepatic Stellate Cell Activation pathway; “Rheumatoid Arthritis” is the Role of Macrophages, Fibroblasts and Endothelial Cells in Rheumatoid Arthritis pathway; and, “Osteoarthritis” includes genes associated with osteoarthritis.

To identify the biological processes potentially important to corneal functions and perturbed in KCN, we used DAVID Bioinformatic Resources and Ingenuity Pathway Analyses, yielding five significant canonical pathways in each patient group (Fig. 4)^52,53^. First, acute phase (tissue injury) responses and IL-6 signaling related DEGs were significant in both patient groups. Second, NRF2-regulated oxidative stress response DEGs were significantly decreased in the KJ samples, with a similar but non-significant trend in KC samples. Third, two networks, often active in arthritis, that essentially regulate immune signals, inflammation and ECM, were decreased in KJ. Finally, PPAR signaling, RAR and ECM-related DEGs were significant in KC. In the following we examine these pathways and their implications for KCN.

Multiple acute phase and tissue injury-response genes were decreased in both patient groups (Fig. 4**)**. These included *CEBPB/TCF5*, a transcription factor that regulates immune and inflammatory responses, and *IL1A*, a pleiotropic cytokine that regulates hematopoiesis, inflammatory and injury responses. Other decreases from this network included *IL6R*, (interleukin 6 receptor subunit alpha), *IL6ST* (a signal transducer for IL6), *SOCS3*, *SOCS8* (suppressors of cytokine signaling), and *SAA1*, encoding an apolipoprotein.

**Figure 5:**
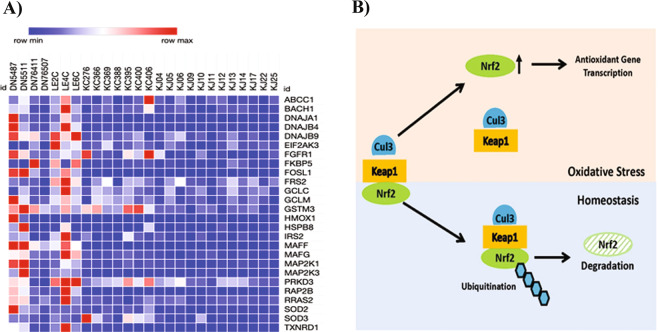
NRF2 target genes significantly altered in KCN corneas. (**A**) Decreased NRF2 target gene expression in KC and KJ RNA seq. (**B**) Mechanism of NRF2 regulation by KEAP1; the latter binds to NRF2 and CUL3 for NRF2-ubiquitination and degradation to maintain low levels of NRF2 under homeostatic conditions. Under oxidative stress, KEAP1 dissociates from NRF2 allowing its increase and upregulation of target genes.

**Figure 6:**
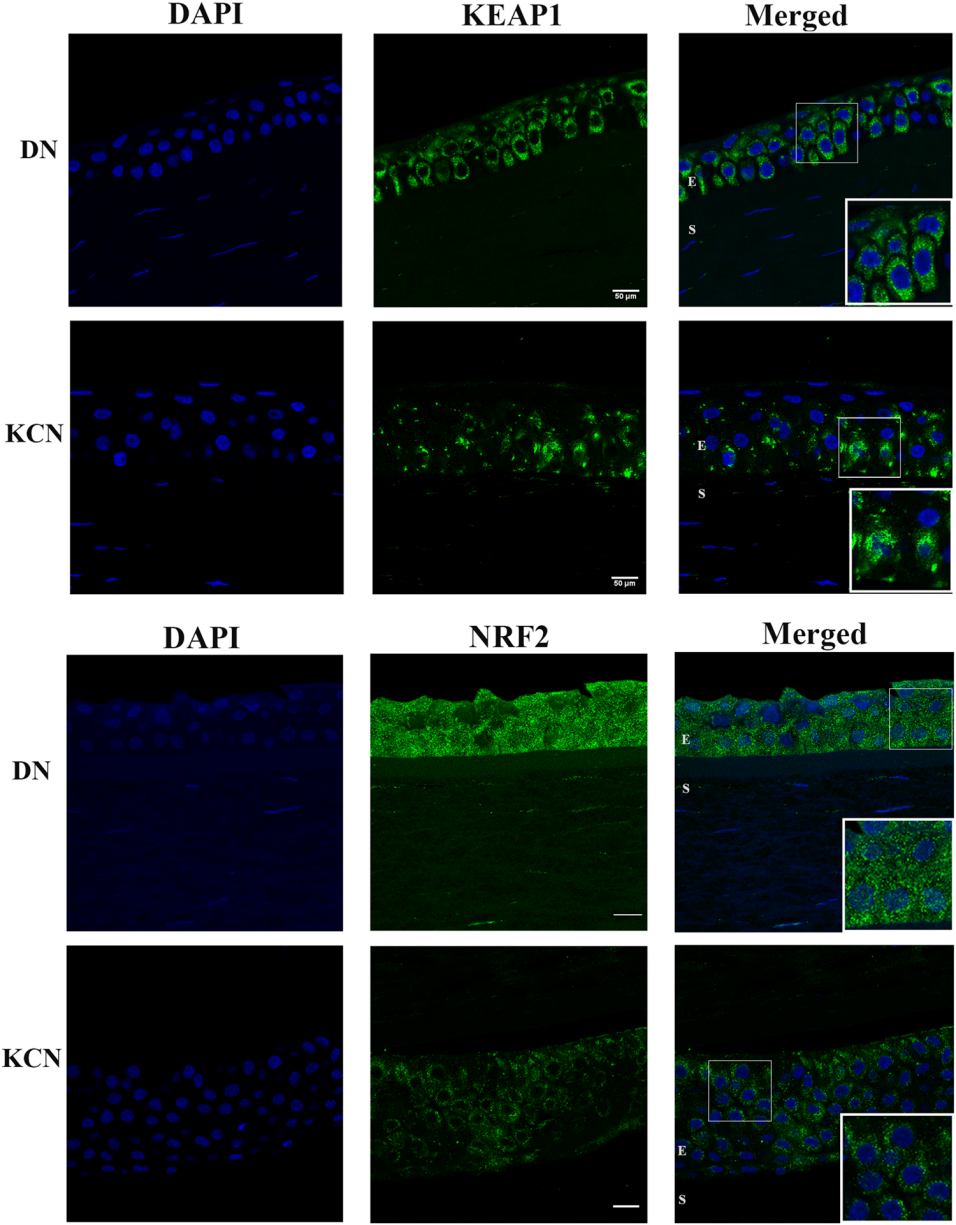
KEAP1 and NRF2 immunostaining in DN and KCN corneas. (**A**) KEAP1 staining is decreased in KCN corneas, with focally increased staining in some basal epithelial cells (inset), whereas in DN corneas KEAP1 shows staining of all epithelial layers (inset). (**B**) NRF2 shows very little to no staining of KCN corneas and these were all cytoplasmic (inset), while DN sections show stronger staining of epithelial cells and some nuclear staining (inset) DAPI nuclear staining shown in blue. IF staining of additional KCN and DN cornea sections are shown in Supplemental Fig. S4. Scale bar: 50 µm.

NRF2 (nuclear factor erythroid2-related factor 2) is a transcription factor that promotes the induction of antioxidants and other components that control reactive oxygen species (ROS) to counteract their harmful effects^54^. NRF2-mediated antioxidant protection is important to the eye and NRF2-boosting treatments are being considered for ocular wound healing and macular degeneration^55^. Multiple antioxidant genes, positively regulated by NRF2, were decreased in KJ, and to a lesser extent in KC (Fig. 5A). These NRF2 target genes include glutathione S-transferase (*GSTM3*), thioredoxin reductase (*TXNRD1*), producers of reducing factors (*GCLC, GCLM*), a transcription regulator (*FOSL1*), chaperone-encoding genes (*DNAJA1, DNAJB4, DNAJB9, UBAP1*) involved in protein-folding, degradation and trafficking, and, *HMOX1*, a heme oxygenase that regulates iron metabolism. NRF2 is itself regulated post-transcriptionally, which may explain why we did not detect it by differential transcriptome analyses. Under homeostatic conditions, NRF2 protein is retained in the cytoplasm and maintained at low levels through its rapid turnover by KEAP1, which mediates interactions with CUL3 leading to ubiquitin proteasomal degradation of NRF2^54^. Elevated ROS leads to disassociation of the KEAP1-NRF2 complex, escape of NRF2 from ubiquitination and degradation (Fig. 5B). A few studies have reported expression of NRF2 target genes, or NRF2 itself in cultured corneal cells, but these did not examine NRF2 in tissue layers of the cornea. Thus, we localized KEAP1 and NRF2 proteins in control DN and keratoconus corneas by immunofluorescence (IF) staining (Fig. 6A,B) in 3 different DN and KCN corneas (Supplemental Fig. S4A,B). We find that both KEAP1 and NRF2 are primarily present in the corneal epithelial layers. In KCN corneas, KEAP1 staining is generally decreased with areas of focal increase in the epithelium (Fig. 6A). IF staining of NRF2 itself is decreased in KCN; in particular DN corneas show more nuclear NRF2 staining (Fig. 6B). Earlier we reported increased KEAP1 and CUL3 proteins in a comparative proteomics of KCN and donor corneas^56^.

### Validations of RNA seq findings

We validated select findings from our transcriptome analyses in three ways: testing RNA expression by qRT-PCR, protein localization and presence by IF staining of donor and keratoconus corneal sections, and against the literature. We selected *ICAM1* (cell adhesion), *TUBB2A*, *TUBB3* (cytoskeletal), *SOD2* (belonging to the NRF2-mediated antioxidant pathway), *RXRA* (nuclear retinoid receptor) and *MFAP3L* (microfibril associated protein3 like) for testing by qRT-PCR because RNA sequencing showed robust expression at FPKM $\ge 5$, which in our experience, allows reliable detection by qRT-PCR (Supplemental Fig. S5 and Table S7). In agreement with the RNA Seq data, qRT-PCR detected elevated *MFAPL3* and decreased *TUBB3* expression in KCN. We detected robust expression of *RXRA* by qPCR, but it was not significantly different between DN and KCN groups. However, *RXRA* by RNA seq was elevated in both the KC (mean 35 FPKM) and the KJ (mean 43 FPKM) patient groups compared to donor samples (17 FPKM). Expression *GAS1* by qRTPCR was slightly increased in KCN, without reaching significance, although by RNA seq, it was markedly increased in both patient groups (Supplemental Table S3). HSP40/DNAJA1, detected as markedly decreased by RNA seq in patients, was decreased by qPCR as well without reaching significance. Taken together, the qPCR data is in general agreement with the RNA seq results.

**Figure 7:**
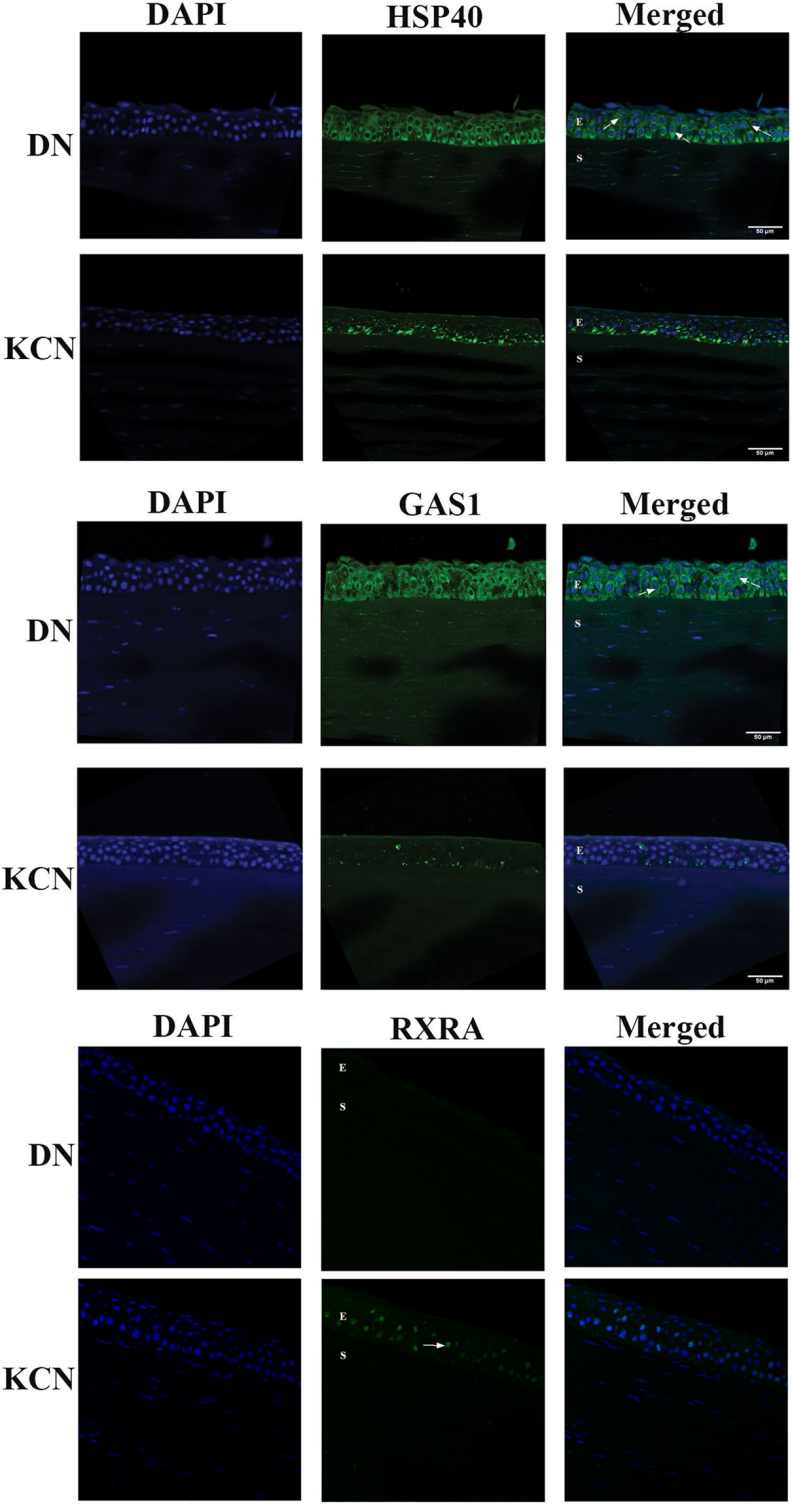
Immunofluorescence staining of selected markers in corneal sections. (**A**) DNAJA1/Hsp40 is weaker in the KCN, with some staining of basal epithelial cells and more uniform staining of all epithelial layers (E) in DN (arrows). (**B**) GAS1 staining was almost non-existent in KCN but show uniform staining of all epithelial layers (arrows) and weak stromal (S) staining in DN. These represent one of two KCN and DN cornea samples. (C) RXRA showed a dramatic increase in nuclear staining in epithelial layers of KCN corneas Scale bar: 50 µm.

We selected *DNAJA1*and *GAS1* for localization of their respective proteins in the cornea by IF staining. DNAJA1 (Hsp40 member a1), unknown to the eye, belongs to the heat shock protein family; other members of this family are known to serve molecular chaperone functions in the lens and the cornea^57^. In agreement with the RNA seq and qRTPCR results, DNA JA1/HSP40 IF staining was decreased in KCN (Fig. 7A). *GAS1* is known to be expressed in the retinal pigment epithelium (RPE) and the cornea, regulates cell growth and eye development, and *Gas1*-null mice which are viable at birth develop microphthalmia^58^. GAS1 shows very little IF staining of KCN corneas (Fig. 7B) compared to uniform strong staining of all donor corneal epithelial layers. While IF staining is not a reliable indication of protein levels, qualitatively dramatically decreased staining of KCN corneal sections contrasts the GAS1 transcript-level increases in both patient groups (Fig. 3). It remains to be seen whether decreased GAS1 staining is due to 1) an actual decrease protein synthesis, or 2) shedding or loss of cell surface or vesicular GAS1 from the corneal layers. IF-staining of RXRA showed a dramatic increase in nuclear staining in epithelial layers of KCN corneas (Fig. 7C). This is consistent with increases in its transcript.

Overall our transcriptome results also agree with findings from earlier studies. For example, *CTGF*, *SMAD7*, *TGFB1* gene expressions were decreased in both KC and KJ samples and were also reported to be decreased as a part of the Hippo, Wnt and TGFß signaling pathways^40^. Consistent with ER stress identified as important by our earlier proteomic studies^30,31,56^, here we detected changes in translation-related factors (*EIF1*, *EIF1B*, *EIF4E*) and proteasomal degradation components (*UBAP1*).

## Discussion

Using deep transcriptome analysis of keratoconus and control corneas from African American and Saudi Arabian patients, we respectively identified 819 and 993 differentially expressed genes. As tissue donation is an extremely rare practice in Saudi Arabia, we selected donor controls of European Ancestry as previously it has been shown that these individuals are genetically closer to Middle Eastern individuals than Africans^43^. In addition, on average donor groups were older than the patient groups, however, there were no age-dependent distrbutionof samples in the principal component analysis. Remarkably, despite differing ancestry and geography (ecology), and the use of generally older control corneas from an eye bank, patient transcriptomes were clustered and clearly separated from control transcriptomes by principal component analysis. Previous transcriptomic analyses have also examined corneas from keratoconus patients^40,41^: in one study, keratoconus corneas were compared to non-keratoconus corneas from patients undergoing cornea grafting for other conditions. These patients had overt inflammation, infection and other eye diseases, and were likely reasons why case versus control corneas were poorly distinguished. The internal consistency of our data, therefore, allowed us to demonstrate decreases in NRF2-mediated oxidative stress and acute phase tissue injury responses as core pathogenic changes in keratoconus in both patient groups. *HMOX1* is an important NRF2-downstream antioxidant target gene that catabolizes heme and resolves iron imbalance and toxicity, and decrease in its transcript in both keratoconus patient groups affirms NRF2 dysfunctions. Reduced *HMOX1* may also contribute to the iron lines or Fleischer’s Ring observed in keratoconus corneas, and the long-held belief of iron imbalance in this disease^3,59^. Additional support for NRF2 impairments in KCN comes from our earlier proteomic study of pooled KCN and donor corneas where NRF2-mediated oxidative stress response proteins were decreased in the epithelium^30^. Given that samples were pooled in that earlier study, we were not fully appreciative of the significance of NRF2 in keratoconus at that time. In a recent proteomic study of individual corneas from keratoconus patients we detected decreases in proteins that regulate complement pathways and tissue injury responses, and other proteins encoded by NRF2-target genes, emphasizing a failure in protective mechanisms in the cornea^56^.

The healthy cornea routinely encounters and resolves reactive oxygen species (ROS) produced in response to restricted nutrient supply, UV exposure, mechanical injury and infections^60^. This ability to dissipate ROS was suspected to be malfunctioning in keratoconus, causing oxidative damage and consequent degenerative changes in corneal cells and the ECM. A “cascade hypothesis” was proposed to tie this oxidative damage to abnormal stromal ECM and keratocyte cell death, as observed in keratoconus^23^. Therefore, it is pathogenetically significant that we have now identified NRF-2 signal disruptions as an important early regulatory step in the poor antioxidant functions in keratoconus. Concordantly, sulforaphane, a NRF-2 activator reduces oxidative stress in an *ex vivo* cell culture model of Fuchs dystrophy^61^ and corneal damage in a rabbit model of corneal thinning^62^.

The two patient groups do harbor some differences as discussed below. First, certain growth factor and inflammation gene-networks are decreased in the Saudi Arabian group, although there are more conjunctivitis cases in this group. The connection between inflammation and keratoconus is not clear however. Historically, KCN is described as a non-inflammatory corneal ectasia, although tear fluid IL-6 increases, TNF-$\alpha$ decreases^63^ and increases^64^ suggest otherwise. The African American cases show significant changes in RAR/Vitamin A signaling, PPAR and ECM-related networks. Interestingly, increased nuclear staining of RXRA in KCN corneas and increases in its transcript in our RNA seq data may tie in with its potential candidate gene stature. Increased transcripts for ECM-related genes, encoding COL1A1, COL3A1 and FN, potentially implicate fibrosis-related changes in the African American group, that we will investigate in a larger set of patients. In a proteomic study of pooled corneas, although on a different set of patients, we noted decreases in some of these same ECM proteins^30^. However, the relationship between transcript level changes in ECM genes and ECM protein accumulation in a tissue is often not direct, as it is influenced by feedback regulation, secretion and correct assembly of ECM proteins, and their degradation and remodeling. A vast body of work has investigated the TGF ß-ECM remodeling axis in the cornea in the context of wound healing and fibrosis^65–69^. Along these lines, we have previously probed TGF ß signals in cultured stromal cells from keratoconus corneas and detected increases in non-canonical TGF ß-SMAD 1/5/8 signals, and poor activation of AKT, that controls cell survival and their metabolic and biosynthetic capabilities^31^. Yet others have reported decreased regulatory SMAD7 in cultured keratoconus stromal cells^70^, which is consistent with our observations that *SMAD7* transcript is decreased in African American cases. Other transcriptomic studies have also reported gene expression decreases in members of the TGF ß-SMAD pathway^40^. Finally, this study shows changes in multiple epithelial genes, providing credence to an existing hypothesis of epithelial damage as a potential driver of stromal degeneration in keratoconus.

Importantly, a small set of genes (*THBS2*, *GAS1*, *OTOP2*, and others) were upregulated across patients in both groups compared to controls. These may yield early biomarkers for keratoconus. In addition, a combination of genes significantly upregulated in the KJ samples only, *S100A8*, *S100A9*, *ADAMTS14*, *LYPD2* and *AOC1* may yield distinguishing biomarkers for subtypes of keratoconus. This will require additional careful phenotyping of disease and its progression, and studies of larger groups for transcript and protein-level changes.

Our study provides three major concepts in keratoconus. First, the degenerative ECM thinning phenotype of the cornea is an outcome of loss of cellular functions that is geared to counteract oxidative stress and tissue injury. Second, the NRF2-regulated gene network has a significant role in this cellular response to oxidative stress in the cornea. Finally, using our approach, analysis of transcriptomic data from different populations and patient groups will help to develop signatures and biomarkers for different subtypes of keratoconus.

## Materials and Methods

### Patient and donor samples

This study was conducted following the Principles of the Declaration of Helsinki on Biomedical Research involving human subjects. We obtained written informed consents from all the participants following a protocol approved by the Institutional Review Board of the Johns Hopkins Medical Institutions, Baltimore, MD and the King Khaled Eye Specialist Hospital (KKESH), Riyadh, Saudi Arabia. Further analyses were performed by study team members and additional guidance of an approved protocol from the Institutional Review Board at NYU Langone Health. A total of 7 and 12 corneas were obtained from patients undergoing cornea transplantation at the Wilmer Eye Institute (WEI) at Johns Hopkins and KKESH, respectively (Supplemental Table S1). The patients from WEI and KKESH were of African American and Saudi Arabian ancestry, respectively, and referred to as KC and KJ throughout the study. Keratoconus diagnosis was performed by trained physicians at both sites and included slit-lamp biomicroscopy, fundus evaluations and corneal topography measurements by Pentacam. Patients at both sites were graded as severe, with central keratometry (K) > 52 diopters (D) or not measurable (WEI) or K > 55D (KKESH). The Lions Eye Institute for Transplant and Research, Florida, provided control corneas, deemed unsuitable for transplants, and without keratoconus or other inflammatory diseases, obtained from deceased individuals of African American (DN) and European American (LE) ancestry. An additional 2 control corneas of unknown ancestry were obtained from WEI (DN373 and DN401). Donor corneas with an intact limbal ring packed in Optisol solution at 4 °C were shipped to Baltimore for these studies, with death to Optisol storage time being approximately 9.5 hours. The donor corneas were received within a week after their procurement and processed immediately for RNA extraction or paraffin-embedding.

### Tissue preparation

Samples received from patients had the central cornea only without peripheral limbal tissue. Central cornea halves received from KKESH patients were snap frozen in liquid nitrogen and stored at −80 °C until shipment on dry ice to WEI for RNA extraction and sequencing. At WEI, patient cornea halves obtained immediately after surgery were placed directly in TRIzol and brought to the laboratory for RNA extraction. The limbal ring was removed from donor corneas, halved, and one half used for RNA extraction.

### RNA extraction, quantification, sequencing and analysis

Total RNA was isolated from individual corneas by homogenization in 1 ml TRIzol Reagent (Life Technologies -15596–026) at room temperature and chloroform-extracted. The RNA in the aqueous phase was precipitated using 0.5 ml of 100% isopropanol and collected by centrifugation at 12,000 g, washed with 75% ethanol and resuspended in RNAse-free water.

A fluorescence-based (Ribogreen, Life technologies) method was used for RNA quantification. cDNA synthesis, fragmentation and library preparation and sequencing were performed by Macrogen Inc. using the TruSeq stranded RNA library kit. A template size check was performed by running the samples on an Agilent Technologies 2100 Bioanalyzer with a DNA chip. The samples were sequenced at 101 bp using the Illumina HiSeq 2500 platform. Raw images were generated using the HiSeq Control software v2.2.38, base calling using an integrated primary analysis software, Real time Analysis v1.18.61.0, and converted into FASTQ files using the Illumina package bcl2fastq v1.8.4. The sequencing library size, total reads, GC content and Q20/Q30 values were comparable for all samples and demonstrated high quality (Supplemental Table S2). After cufflink quantification (file “isoforms.fpkm_tracking” and file “genes.fpkm_tracking”), we obtained transcript-level and gene-level expression values (Table 1); expressed transcripts were used to obtain summed FPKM (fragments per kilobase of transcripts per million mapped reads) counts per gene.

Alignment of reads against the human reference genome hg19 was performed using the STAR 2.6.0a software. During alignment, we removed non-canonical junctions (option:–outFilterIntronMotifs RemoveNoncanonical) and generated XS strand attributes for splice alignments (option:–outSAMstrandField intronMotif). Gene expression quantification was completed using Cufflinks 2.2.1^45^ with the following settings: 1) all annotated rRNA and mitochondrial transcripts were ignored (option: -mask-file); 2) bias detection and correction algorithm was enabled (option: –frag-bias-correct); 3) reads mapping to multiple locations in the genome were weighted during an initial estimation procedure (option: –multi-read-correct); 4) UCSC hg19 annotation file was supplied (option: –GTF-guide). We first checked for contamination, adaptor sequences, or other overrepresented sequences in the raw reads (FASTQ files) of all 36 sequenced samples by FastQC 0.11.7 and found no evidence of these. Next, differential expression analysis was conducted using Cuffdiff with bias correction and multi-reads corrections^44^. Genes identified as significantly different were confirmed by reanalysis using DESeq2 (version: 1.24.0) with default parameters^46^. In DESeq2, for final statistical significance of a gene, we used Benjamini-Hochberg adjusted p ≤ 0.01, absolute fold change ≥2, and FPKM ≥5 in at least one group.

FPKM value for summed transcripts was used as each gene’s FPKM value. We defined significant differential expression by q value $\ge$ 0.01 (FDR adjusted p), |fold change | $\ge$2, and FPKM $\ge$5 in control or patient groups. Next, we used Ingenuity Pathway Analysis (IPA) software (Qiagen) on these significant differentially expressed genes (DEG) to identify enriched biological pathways and networks among these genes.

For Principal Component Analysis (PCA) of sequenced samples, we used two definitions: a) genes expressed at FPKM $\ge$5 in *any one* sample, and b) genes expressed at FPKM $\ge$5 in *all* samples. PCA was performed using the “prcomp()” function in R “stats” Package version 3.5.0 after expression values were log_2_(FPKM + 1) transformed. The coordinates for each sample in the principal components (PC1 and PC2) were used to calculate the Euclidian distance between samples by the function “pointDistance()” in R “raster” Package version 3.0–2.

### Gene expression assays by qRT-PCR

cDNA was prepared from each RNA sample using a cDNA Reverse transcription kit (Biorad). Each cDNA (20 ng) was subjected to qRT-PCR using Applied Biosystems TaqMan assays for selected genes on a One Step Plus instrument (Applied Biosystems). The number of cycles (Ct) needed to reach the midpoint of the linear phase was noted and all observations were normalized against the housekeeping gene *GAPDH*.

### Immunofluorescence (IF) staining

Paraffin-embedded cornea sections were stained with H&E (NYU School of Medicine Center for Biospecimen Research & Development). For immunofluorescence (IF), slides were blocked with 10% animal serum or BSA in PBS, followed by overnight incubation at 4 °C in primary antibody, washed three times with Tris-buffered saline, incubated with a secondary antibody for 2 hours and the nuclei counterstained with DAPI. The following primary antibodies were used from Biorbyt Research Products: NRF2 (orb128433, 1:50), KEAP1 (orb48426, 1:100), Hsp40 (orb520080, 1:100), and GAS1 (orb414757, 1:100) and RXRA antibody were used from Novus Biologicals (NBP2–75653, 1:50). A fluorescently conjugated anti-rabbit secondary antibody (Invitrogen, Cat# A21206) was used at a concentration of 5μg/ml. Images were acquired with a Zeiss LSM 700 microscope.

## Supplementary Information

Supplementary Information.

Supplementary Information2.

Supplementary Information3.

Supplementary Information4.

Supplementary Information5.

Supplementary Information6.

Supplementary Information7.

Supplementary Information8.

Supplementary Information9.
