# Supplementary material for: RNA sequencing of corneas from two keratoconus patient groups identifies potential biomarkers and decreased NRF2-antioxidant responses
Source: Sci Rep. 2020 Jun 18;10:9907. doi: 10.1038/s41598-020-66735-x (PMC7303170; doi:10.1038/s41598-020-66735-x)
Supplement: Supplementary file 1 — Supplementary Information. [file 41598_2020_66735_MOESM1_ESM.pdf]

# RNA sequencing of corneas from two keratoconus patient groups identifies potential biomarkers and decreased NRF2-antioxidant responses

Vishal Shinde PhD [1#], Nan Hu MS [1#], Alka Mahale PhD [2], George Maiti PhD [1], Yassine Daoud MD [3], Charles G. Eberhart MD [4], Azza Maktabi MD [2], Albert S. Jun MD [3], Samar A. Al-Swailem MD [2], and Shukti Chakravarti [1,5] PhD *

Running Head: Keratoconus Transcriptomes of two distinct patient groups

***Corresponding author:**

Shukti Chakravarti, Ph.D.
Department of Ophthalmology
Department of Pathology
NYU Langone Health
T: 646-501-8464 (office)
T: 646-501-8470 (Lab)
E: Shukti.Chakravarti@nyulangone.org

[1] Department of Ophthalmology, NYU Langone Medical Center, New York, NY, USA
[2] King Khaled Eye Specialist Hospital, Riyadh, Saudi Arabia
[3] Wilmer Eye Institute, Johns Hopkins University School of Medicine, Baltimore, MD, USA
[4] Ophthalmology and Oncology Johns Hopkins University School of Medicine, Baltimore, MD, USA
[5] Department of Pathology, NYU Langone Medical Center, New York, NY, USA
[#] These authors contributed equally

**Supplemental Fig. S1**

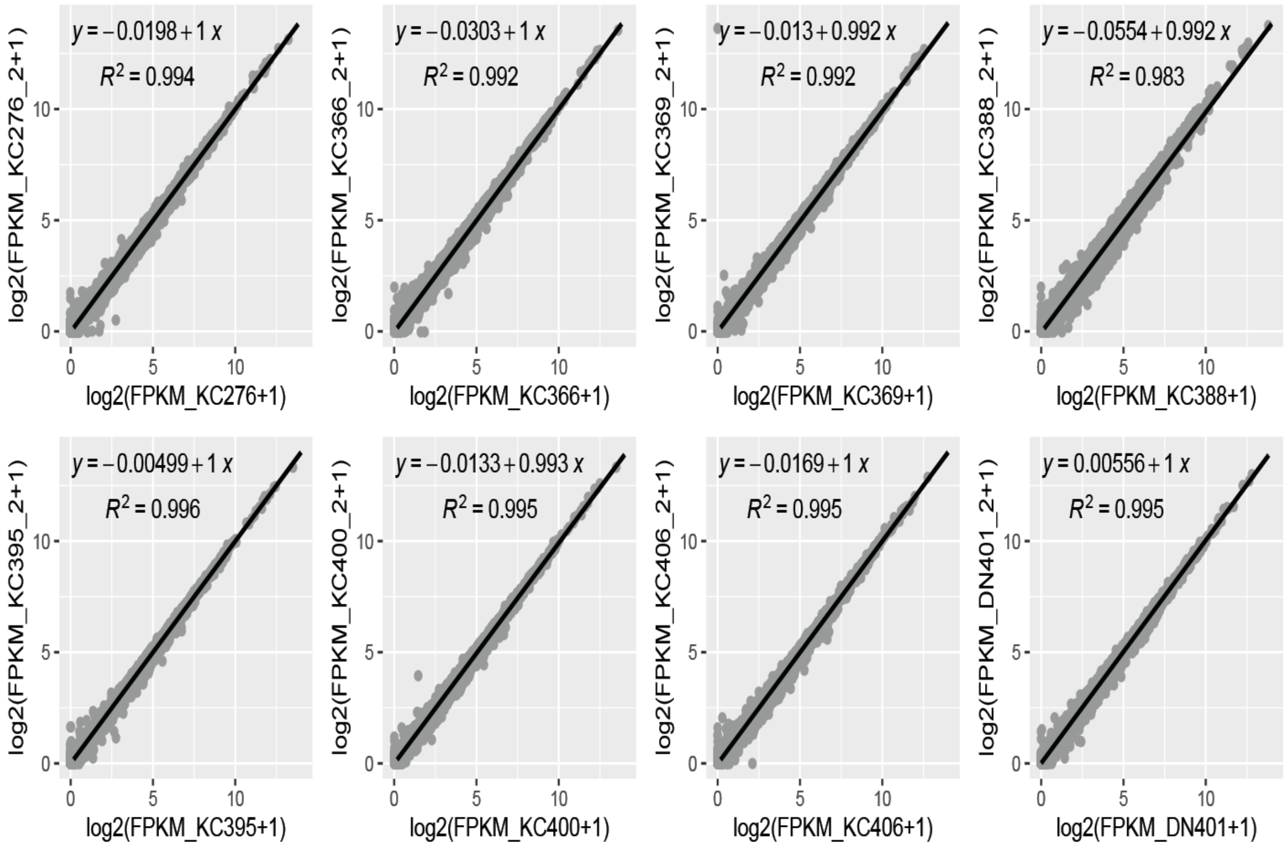


Fig.S1A: Technical replicates of RNA sequencing data are highly reproducible. Eight RNA samples from the Baltimore group were sequenced twice. The X and Y axes represent $\log_2$ transformed (FPKM+1) value of the first and the second sequence data, respectively, and shows high reproducibility between technical replicates.

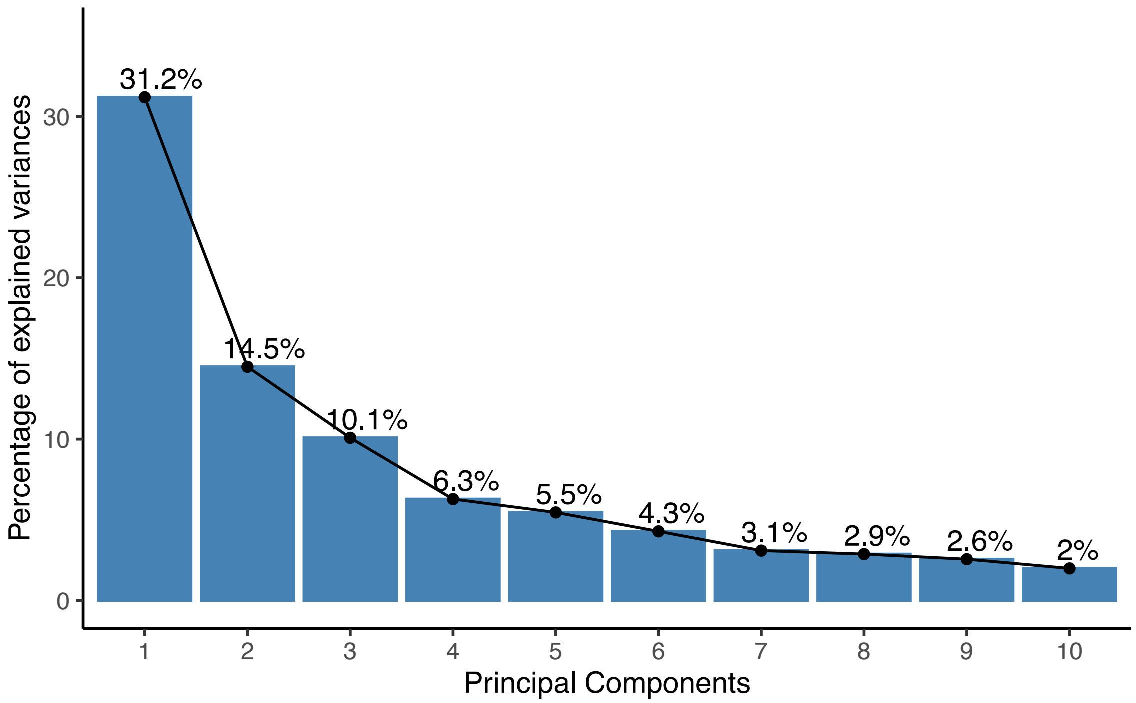


Fig. S1B: A Scree plot showing the fraction of total variance as explained by each Principal Component.

**Supplemental Fig S2**

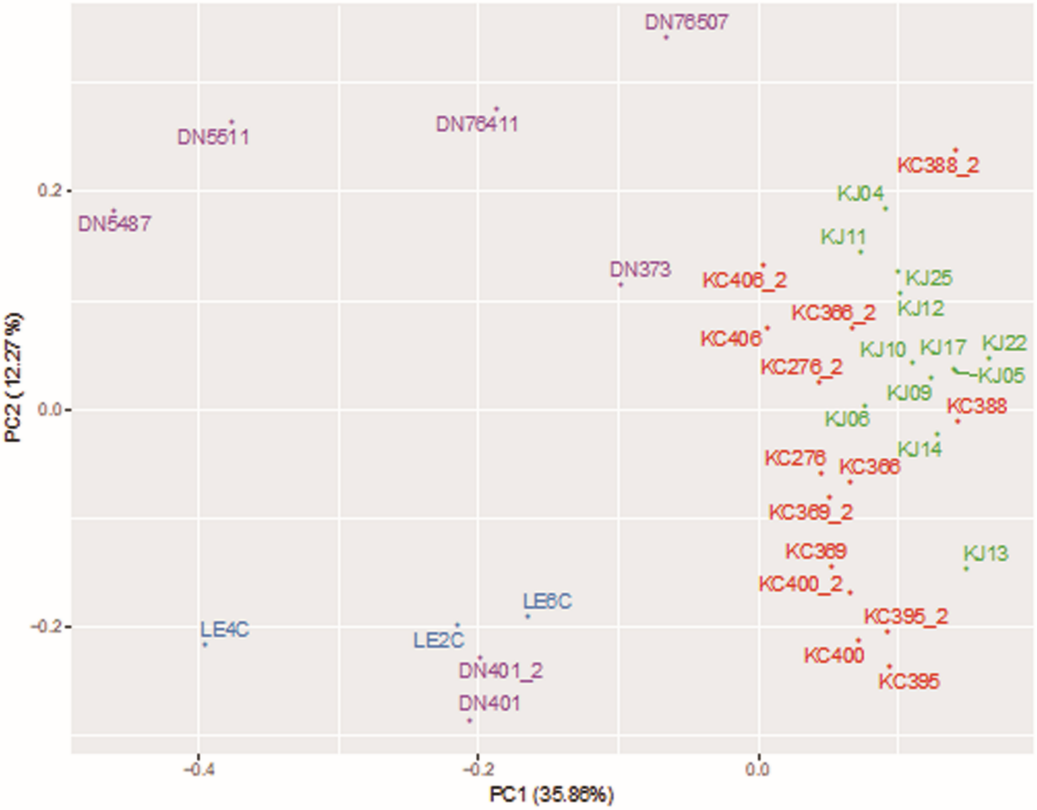


Principal component analysis (PCA) of Keratoconus and controls. 4,787 genes at FPKM≥ 5 in all samples were used. The major separation along PC1 was between KCN and controls. KC and KJ samples cluster together along PC1. African American donor (DN) and KC patient samples are labeled purple and red, respectively; Caucasian donors (LE) are in blue and Middle Eastern patients (KJ) in green. Euclidean distance between samples in a pairwise comparison of samples in PC1 and PC2 is shown in Supplemental Table S3.

**Supplemental Fig S3**

**A)**

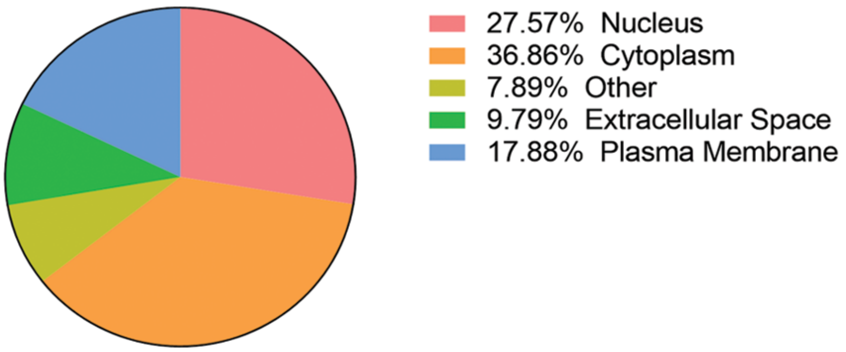


**B)**

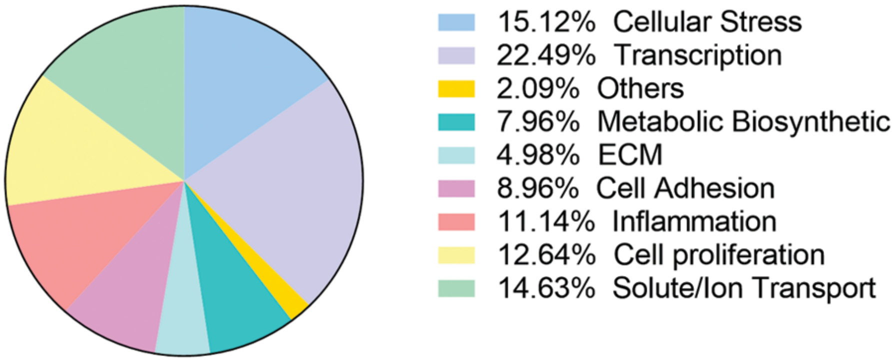


**Fig. S3 (A).** Differentially expressed gene (DEG) classification based on protein localization. All DEGs (819 in KC and 993 in KJ) are categorized based on the Ingenuity Pathway Analysis (IPA) annotated location of encoded proteins. The percent of each category is the average of KC and KJ. **(B)** DEG classification by function using the DAVID Bioinformatics Resource 6.8, with additional manual annotation.

## Supplemental Fig S4

## Fig S4A

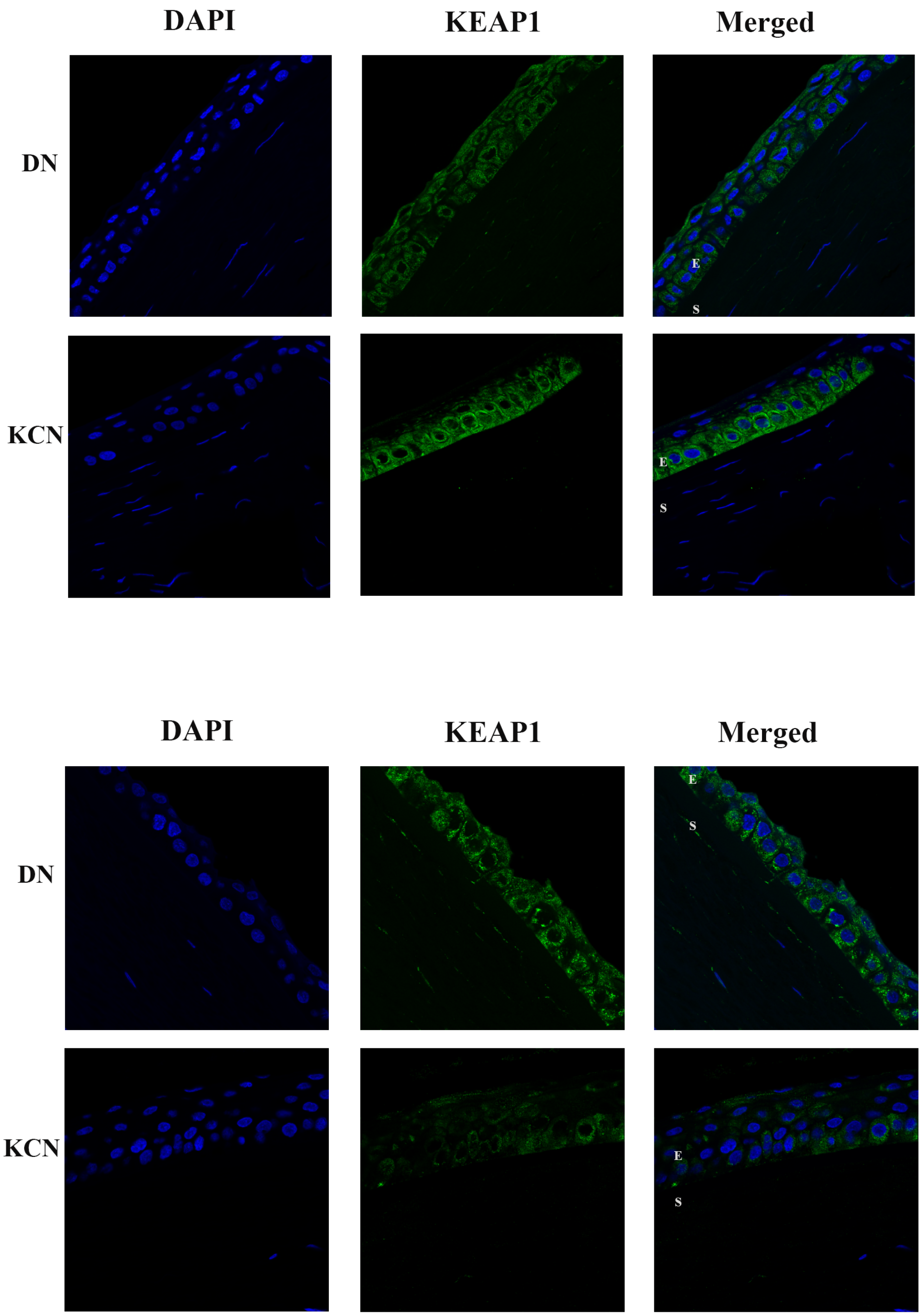


**Fig S4 (A)** KEAP1 immunostaining in 2 individual DN and KCN corneas.

**Fig S4B**

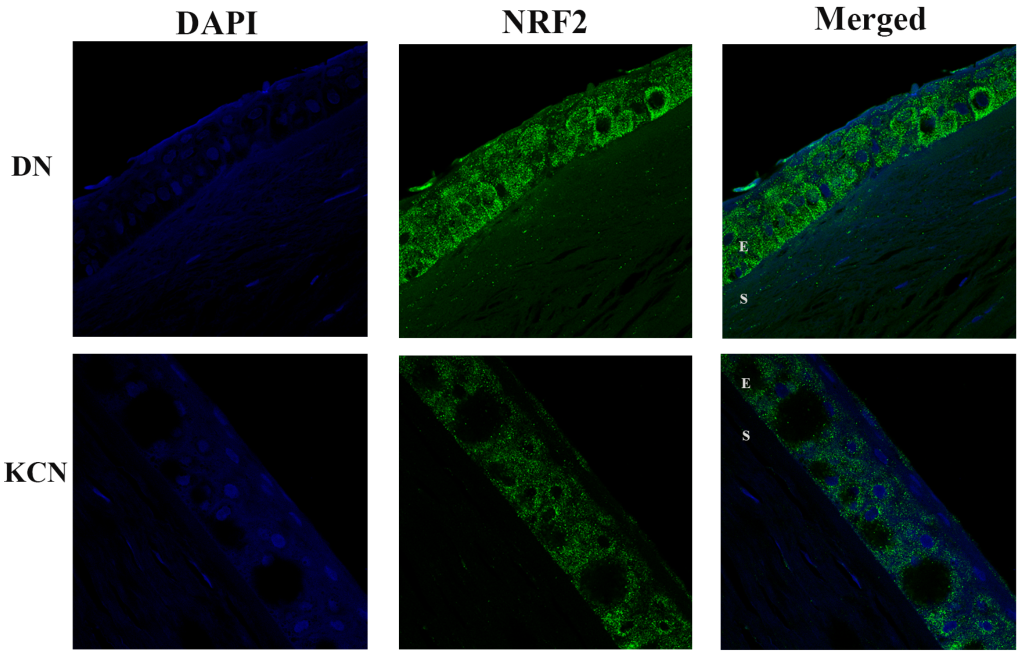


**Fig S4 (B)** Immunostaining of NRF2 in DN and KCN corneas.

## Supplemental Fig S5

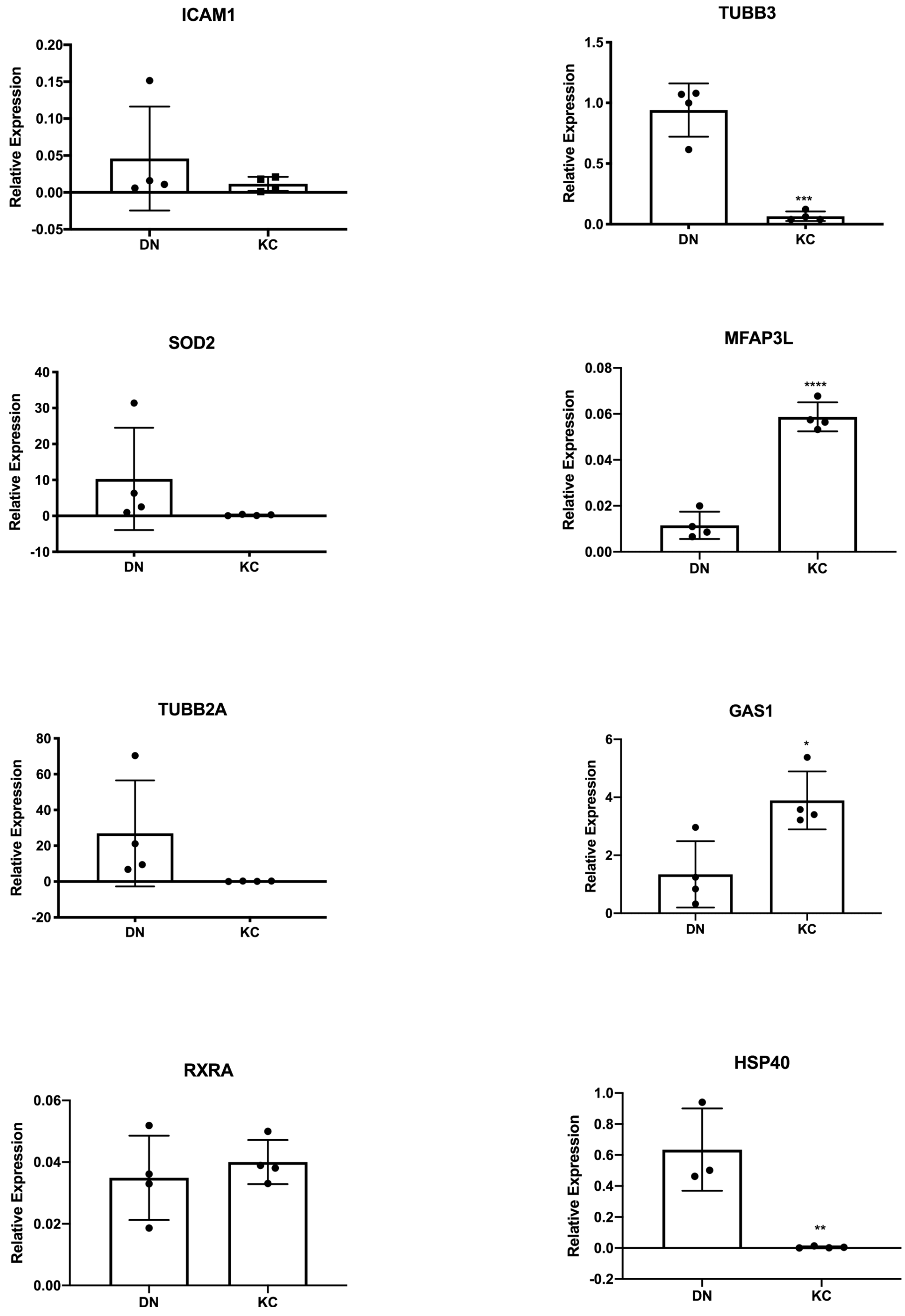


The expression of selected genes in 4DN and 4KC human cornea was measured by qRTPCR using TaqMan™ primers. Relative expression ($2^{-\Delta CT}$) normalized to GAPDH was plotted using Graphpad Prism v7. Data are shown as means ± S.E.M, the statistical significance was measured using unpaired t- test; $*P < 0.05$, $**P < 0.01$. The list of Taqman primer is provided in Supplemental Table S7.

**Supplemental Fig S6**

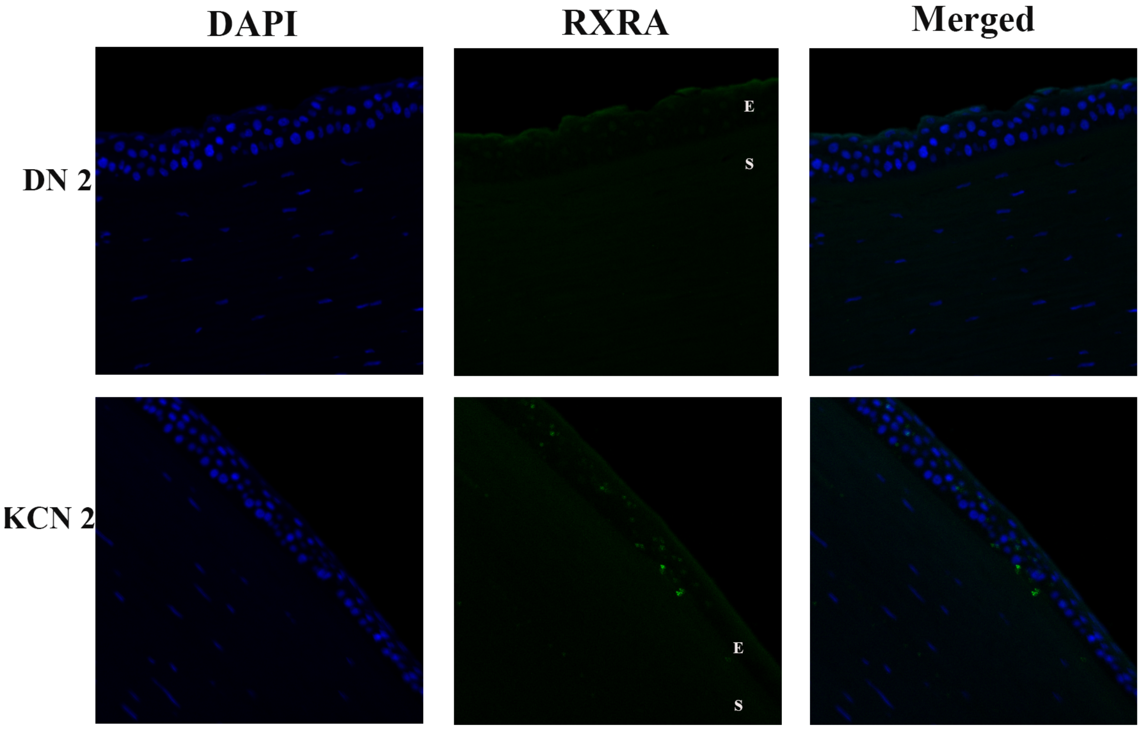


**Fig S6** RXRA immunostaining in individual DN and KCN corneas.
