# Supplementary material for: RNA sequencing of corneas from two keratoconus patient groups identifies potential biomarkers and decreased NRF2-antioxidant responses
Source: Sci Rep. 2020 Jun 18;10:9907. doi: 10.1038/s41598-020-66735-x (PMC7303170; doi:10.1038/s41598-020-66735-x)
Supplement: Supplementary file 3 — Supplementary Information3. [file 41598_2020_66735_MOESM3_ESM.pdf]

Supplemental Table S1: Patient demographics and clinical characteristics.*

Sample ID	Group	Age in years	Sex	Ancestry	Atopy/Allergy	Disease Severity
LE2C	control	64	F	EA	NA	NA
LE4C	control	58	M	EA	NA	NA
LE6C	control	64	F	EA	NA	NA
DN5487	control	25	F	AA	NA	NA
DN5511	control	52	M	AA	NA	NA
DN76411	control	64	F	AA	NA	NA
DN76507	control	75	M	AA	NA	NA
KC276	case	24	M	U	Absent	Severe
KC366	case	29	F	AA	Absent	Severe
KC369	case	46	F	AA	Absent	Severe
KC388	case	20	M	AA	Eczema, hay fever, allergies	Severe
KC395	case	17	M	AA	Absent	Severe
KC400	case	69	M	AA	Asthma	Severe
KC406	case	48	F	AA	Absent	Severe
KJ04	case	20	M	ME	Absent	Severe
KJ05	case	21	M	ME	Vernal keratoconjunctivitis	Severe
KJ06	case	33	F	ME	Absent	Severe
KJ09	case	27	F	ME	Seasonal allergic conjunctivitis	Severe
KJ10	case	23	M	ME	Absent	Severe
KJ11	case	18	F	ME	Vernal keratoconjunctivitis	Severe
KJ12	case	25	M	ME	Vernal keratoconjunctivitis	Severe
KJ13	case	27	F	ME	Absent	Severe
KJ14	case	29	M	ME	Absent	Severe
KJ17	case	36	F	ME	Absent	Severe
KJ22	case	36	M	ME	Absent	Severe
KJ25	case	32	F	ME	Absent	Severe
Samples not used in differential expression analysis						
DN373	control	U	U	U	NA	NA
DN401	control	69	F	U	NA	NA

***Sample sources:** DN and LE are control samples from the Lions Eye Institute for Transplant and Research, Florida; DN373 and DN401 are control samples from the Wilmer Eye Institute, Johns Hopkins University School of Medicine, Baltimore, USA; KC are Keratoconus patient samples from the Wilmer Eye Institute, Johns Hopkins University School of Medicine, Baltimore, USA; and, KJ are Keratoconus patient samples from the King Khalid Eye hospital, Riyadh, Saudi Arabia. **Abbreviations:** Case/control: Keratoconus/disease-free subjects; F/M: female/male; EA/AA/ME: European American/African American/Middle Eastern; NA: not applicable; U: unknown.
