# Supplementary material for: RNA sequencing of corneas from two keratoconus patient groups identifies potential biomarkers and decreased NRF2-antioxidant responses
Source: Sci Rep. 2020 Jun 18;10:9907. doi: 10.1038/s41598-020-66735-x (PMC7303170; doi:10.1038/s41598-020-66735-x)
Supplement: Supplementary file 4 — Supplementary Information4. [file 41598_2020_66735_MOESM4_ESM.pdf]

Supplemental Table S2: Corneal samples RNA yield and quality.

Sample ID	RNA yield (μg)	RIN #	Library size (bp)	Total # reads	GC (%)	Q20 (%)	Q30 (%)
LE2C	3.41	9.3	284	60,869,200	49.48	96.08	93.28
LE4C	1.17	8.7	283	73,679,166	48.74	96.16	93.42
LE6C	1.12	9.3	308	84,290,920	49.59	95.99	93.13
DN5487	1.90	7.3	283	84,275,090	51.34	95.29	91.92
DN5511	2.95	7.6	279	68,279,876	52.69	95.57	92.36
DN76411	3.98	8.0	270	63,343,026	52.79	95.80	92.72
DN76507	6.25	6.9	275	61,435,316	53.23	95.59	92.37
KC276_2	1.12	8.5	279	59,776,822	52.49	95.47	92.20
KC366_2	0.72	7.9	277	61,019,468	51.68	95.76	92.66
KC369_2	1.01	8.8	275	62,422,154	51.39	95.34	91.98
KC388_2	1.42	9.7	280	63,850,040	52.90	95.63	92.44
KC395_2	0.94	7.7	286	73,387,096	51.10	94.96	91.43
KC400_2	0.91	8.8	281	64,445,560	52.20	95.33	91.97
KJ04	2.15	8.0	284	70,504,602	50.45	95.18	91.76
KJ05	3.82	8.2	283	61,149,826	49.97	95.51	92.32
KJ06	2.90	8.7	279	57,686,834	49.58	95.70	92.64
KJ09	0.45	8.6	279	64,356,492	48.67	95.92	93.03
KJ10	1.52	7.8	279	63,822,798	49.40	95.98	93.11
KJ11	3.70	5.2	282	76,409,238	50.44	95.38	92.14
KJ12	0.36	7.3	279	64,008,804	49.27	95.78	92.79
KJ13	1.68	8.5	279	77,471,920	49.43	95.75	92.74
KJ14	1.80	7.8	286	59,141,526	49.85	95.80	92.83
KJ17	0.73	8.0	286	63,144,364	49.19	95.88	92.95
KJ22	0.85	5.2	283	60,963,934	48.72	95.65	92.57
KJ25	0.88	7.7	284	57,934,452	49.36	95.64	92.56
Samples not used in differential expression analysis							
DN373	1.20	7.5	286	72,562,042	49.25	95.33	92.04
DN401	2.16	8.2	279	72,695,846	50.23	95.60	92.47
DN401_2	2.16	8.2	276	60,527,396	52.49	95.72	92.60
KC406	0.76	8.9	279	63,544,028	52.38	95.46	92.19
KC276	1.12	8.5	282	55,493,880	49.25	95.35	92.09
KC366	0.72	7.9	279	77,267,478	48.97	95.64	92.54
KC369	1.01	8.8	291	72,489,898	48.79	95.11	91.68
KC388	1.42	9.7	285	62,251,528	49.84	95.46	92.24
KC395	0.94	7.7	281	65,026,054	49.20	94.82	91.23
KC400	0.91	8.8	277	74,484,644	49.58	95.18	91.80
KC406	0.76	8.9	281	74,740,710	49.17	95.42	92.20
