# Supplementary material for: RNA sequencing of corneas from two keratoconus patient groups identifies potential biomarkers and decreased NRF2-antioxidant responses
Source: Sci Rep. 2020 Jun 18;10:9907. doi: 10.1038/s41598-020-66735-x (PMC7303170; doi:10.1038/s41598-020-66735-x)
Supplement: Supplementary file 8 — Supplementary Information8. [file 41598_2020_66735_MOESM8_ESM.pdf]

Supplemental Table S6: The ten most increased and decreased DEG.*

A. Ten most increased in KC samples

Gene symbol	Gene name	KC	KJ
LRRC15	Leucine Rich Repeat Containing 15	+	-
COL3A1	Collagen Type III Alpha 1 Chain	+	-
COL1A1	Collagen Type I Alpha 1 Chain	+	-
TMEM119	Transmembrane Protein 119	+	-
MMP11	Matrix Metallopeptidase 11	+	-
TNC	Tenascin C	+	-
C3	Complement C3	+	-
THBS2	Thrombospondin 2	+	+
TNFRSF11B	TNF Receptor Superfamily Member 11b	+	-
FN1	Fibronectin 1	+	-

B. Ten most increased in KJ samples

Gene symbol	Gene name	KC	KJ
S100A9	S100 Calcium Binding Protein A9	-	+
S100A8	S100 Calcium Binding Protein A8	-	+
AOC1	Amine Oxidase Copper Containing 1	-	+
MAB21L1	Mab-21 Like 1	-	+
LGALS9C	Galectin 9C	-	+
CLIC3	Chloride Intracellular Channel 3	-	+
ADAMTS14	ADAM Metallopeptidase with Thrombospondin Type 1 Motif 14	-	+
KRT78	Keratin 78	-	+
LYPD2	LY6/PLAUR Domain Containing 2	+	+
LCN2	Lipocalin 2	-	+

C. Ten most decreased in KC and KJ samples

Gene symbol	Gene name	KC	KJ
HSPA6	Heat Shock Protein Family A (Hsp70) Member 6	+	+
IL1A	Interleukin 1 Alpha	+	+
CA3	Carbonic Anhydrase 3	+	+
PLAUR	Plasminogen Activator, Urokinase Receptor	+	+
FOSL1	FOS Like 1, AP-1 Transcription Factor Subunit	+	+
ATF3	Activating Transcription Factor 3	+	+
HMOX1	Heme Oxygenase 1	+	+
CXCL1	C-X-C Motif Chemokine Ligand 1	+	-
DUSP5	Dual Specificity Phosphatase 5) is a Protein Coding gene	+	+
SERPINB2	Serpin Family B Member 2) is a Protein Coding gene	+	+
CXCL8	C-X-C Motif Chemokine Ligand 8	-	+
LIF	LIF Interleukin 6 Family Cytokine	-	+
NR4A1	Nuclear Receptor Subfamily 4 Group A Member 1	+	+
AREG	Amphiregulin	+	+
SLC2A3	Solute Carrier Family 2 Member 3	+	+

*+ and – refer to statistically significant and not significant results in each sample set.
