# Supplementary material for: RNA sequencing of corneas from two keratoconus patient groups identifies potential biomarkers and decreased NRF2-antioxidant responses
Source: Sci Rep. 2020 Jun 18;10:9907. doi: 10.1038/s41598-020-66735-x (PMC7303170; doi:10.1038/s41598-020-66735-x)
Supplement: Supplementary file 9 — Supplementary Information9. [file 41598_2020_66735_MOESM9_ESM.pdf]

Supplemental Table S7: List of TaqMan Primers

	Gene	Catalog Number	Vendor
1	ICAM1	Hs00164932	Thermo Fisher Scientific
2	SOD2	Hs00167309	Thermo Fisher Scientific
3	TUBB3	Hs00801390	Thermo Fisher Scientific
4	TUBB2A	Hs00742533	Thermo Fisher Scientific
5	RXRA	Hs01067640	Thermo Fisher Scientific
6	MFAP3L	Hs04189224	Thermo Fisher Scientific
7	HSP40	Hs01039243_g1	Thermo Fisher Scientific
8	GAS1	Hs04997460_s1	Thermo Fisher Scientific
